# An analysis of the global pharmacy workforce capacity

**DOI:** 10.1186/s12960-016-0158-z

**Published:** 2016-10-10

**Authors:** Ian Bates, Christopher John, Andreia Bruno, Pamela Fu, Shirin Aliabadi

**Affiliations:** 1 FIP Collaborating Centre, UCL School of Pharmacy, 29-39 Brunswick Square, London, WC1N 1AX United Kingdom; 2 Global Pharmacy Workforce Observatory, Royal Pharmaceutical Society, 66-68 East Smithfield, London, E1W 1AW United Kingdom; 3 International Pharmaceutical Federation (FIP), 2517 JP The Hague, The Netherlands

**Keywords:** Pharmacy workforce, Global, Capacity, Healthcare

## Abstract

**Background:**

The World Health Organization (WHO) estimates that there is a global healthcare workforce shortage of 7.2 million, which is predicted to grow to 12.9 million by 2035.

Globally, people are living longer with multiple co-morbidities and require increased access and use of medicines. Pharmacists are a key component of the healthcare workforce, and in many countries, pharmacists are the most accessible healthcare profession. This paper identifies key issues and current trends affecting the global pharmacy workforce, in particular workforce distribution, country economic status, capacity, and workforce gender balance.

**Methods:**

National professional pharmacy leadership bodies, together with other contacts for professional bodies, regulatory bodies, and universities, were approached to provide country-level data on pharmacy workforce. A descriptive and comparative analysis was conducted to assess each country’s pharmacy workforce.

**Results:**

A total of 89 countries and territories responded to the survey. To standardise the capacity measure, an analysis of the population density of pharmacists (per 10 000 population) was performed. The sample mean was 6 pharmacists per 10 000 population (*n* = 80). There is considerable variation between the surveyed countries/territories ranging from 0.02 (Somalia) to 25.07 (Malta) pharmacists per 10 000 population. African nations have significantly fewer pharmacists per capita. Pharmacist density correlates with gross national income (GNI) and health expenditure. The majority of pharmacists are employed in community settings, followed by hospital, industry-related, academia, and regulation. There is a greater proportion of females in the pharmacy workforce globally, with some WHO regions showing female representation of more than 65 % with an increasing trend trajectory.

**Conclusions:**

Pharmacy workforce capacity varies considerably between countries and regions and generally correlates with population- and country-level economic indicators. Those countries and territories with lower economic indicators tend to have fewer pharmacists and pharmacy technicians; this has implications for inequalities regarding access to medicines and medicine expertise.

## Background

The global healthcare workforce is undergoing dramatic changes with increasing national populations, longer life expectancies, increasing healthcare costs, and rapidly growing demands for health services and burden of chronic diseases affecting both the scale and scope of practice. The World Health Organization (WHO) estimates that there is a global healthcare workforce shortage of 7.2 million, which is predicted to grow to 12.9 million by 2035 [1]. Prior to this in September 2000, the United Nations launched the Millennium Development Goals (MDGs) to combat poverty, hunger, disease, illiteracy, environmental degradation, and discrimination against women by 2015 [2]. It soon became apparent that the global health workforce crisis was one of the greatest constraints in delivering the MDGs. In 2006, a WHO report emphasised the need for direct investment in the training of healthcare workers as well as more efficient use of their skills [3]. In the years following, further reports have highlighted shortages in the healthcare workforce and the effect on implementation of primary care, efficient and equitable use of financial resources, and expansion of health services [4, 5]. Without a doubt, this has focussed global attention on the sustainable evolution of the provision of healthcare services.

Globally, people are living longer with multiple co-morbidities and are requiring increased access and use of medicines. Pharmacists are a key component of the healthcare workforce, and in many countries, pharmacists are the most accessible healthcare profession. Pharmacists play an important role in the delivery of healthcare services since they are involved in community and hospital environments, as well as academia, research, and regulation. However, pharmacist workforce shortages have been reported in all sectors [6]. Alongside the increased demand for the global healthcare workforce, the pharmacy profession itself is undergoing dynamic change with more of a focus on patient-centred care, clinical decision-making on medicine use, and interprofessional collaboration. Whilst pharmacists are trusted and accessible healthcare professionals, it is important to monitor how the pharmacy workforce is changing. These changes will affect the planning of the delivery of healthcare services. There is an imperative to understand the current trends in the global pharmacy workforce and the implications of these trends on the future supply of pharmacists. Only then can it be decided how and what measures are required in order to balance the demand versus supply of pharmacists to help improve the global healthcare workforce.

The objective of this paper is to recognise the key issues and current trends affecting the global pharmacy workforce. In particular, a focus on workforce distribution, country economic status, capacity building, and workforce gender balance. The survey was conducted by the International Pharmaceutical Federation (FIP) Education Initiative (FIP*Ed*); the data has been validated with respondents and shared with the WHO Human Resources for Health. Future articles will explore the data collated on pharmacy education and pharmacy technicians in more depth.

## Methods

The study was based on a survey of national agencies (professional leadership bodies, health workforce regulators, ministries) developed in collaboration with the FIP Collaborating Centre, University College London School of Pharmacy and the FIP Education Initiative (FIP*Ed*). The survey gave a background to the reasons for collecting the data and was composed of 42 questions which sought data relating to pharmacy education, workforce (absolute numbers of pharmacists and pharmacy technicians), and relevant regulations for both pharmacists and pharmacy technicians and was available in English, French, and Spanish. It was conducted by sending a document version of the survey via email to contacts derived from FIP and was conducted over the time period 2012–2013, with repeat follow-up for non-responders. Responses were collated on an Excel® spreadsheet for further analysis.

The analysis used multiple data sources including national population- and country-level economic indicators (as reported by the World Bank [7]). WHO regional comparisons were obtained from information on the WHO website. In summary, information about pharmacy education, workforce, and regulations was obtained via the data collection survey whereas secondary information, e.g. economic indicators and national populations were obtained online from the official data sources described above.

The dataset was cleaned and checked with respondents (who reported information about pharmacy education, workforce numbers, and regulations) before being prepared for analysis using SPSS Statistics v22. The statistical methods used were descriptive (frequencies, central tendencies) and regression (correlation and linear). Limitations include a reliance on published data and secondary sources for some national data and an assumption that the data uses head counts of licensed pharmacists with no differentiation between part-time and full-time workers (analysis was not conducted using whole or full-time equivalents).

Statistical analysis was performed to assess the workforce of each country’s pharmacist and pharmacy technician workforce size and capacity standardised by population, standardised density of pharmacies (hence possible accessibility to medicines), associations with country economic status, gender distribution of pharmacists, and workforce distribution by pharmacy sector.

## Results

A total of 89 countries and territories responded to the data collection survey. Table 1 shows national respondent frequencies by WHO regions. The response rate is shown for each WHO region against the percentage of all WHO member states, for example, African countries represented 25 % of respondents to the survey and account for 23.7 % of all WHO member states. Due to small workforce numbers related to the Pacific Island Countries (PICs), these countries were aggregated into a single PIC case entity for the analysis. The final usable data after verification was 81 cases presented here. The total case load represents around three quarters of the current world population and around half of all United Nations (UN) member states. The analysis presented should be interpreted with the caveat that the data was generalised at the country level (in that pharmacist density correlated to national economic indicators) and based on the best available validated data collated by the FIP*Ed* team.

**Table 1:** Respondent frequencies by sample and WHO region

WHO region	Respondents in sample	Percent	All available WHO member states	Percent
Africa	19	21.4	46	23.7
Americas	8	9.0	35	18.0
Eastern Mediterranean	6	6.7	22	11.3
Western Pacific	23	25.8	27	13.9
South East Asia	6	6.7	11	5.7
Europe	27	30.3	53	27.3
Total	89	100.0	194	100.0

Collectively, the countries in this sample represent around 2.5 million pharmacists and 1.4 million pharmacy technicians. Comparing the pharmacist and pharmacy technician groups with country/territory population, there are correlations (*R* ^2^ = 0.36 *P* < 0.0001 and *R* ^2^ = 0.45 *P* < 0.0001, respectively). African countries tend to have a greater tendency for fewer pharmacists and pharmacy technicians per country population.

**Fig. 1:**
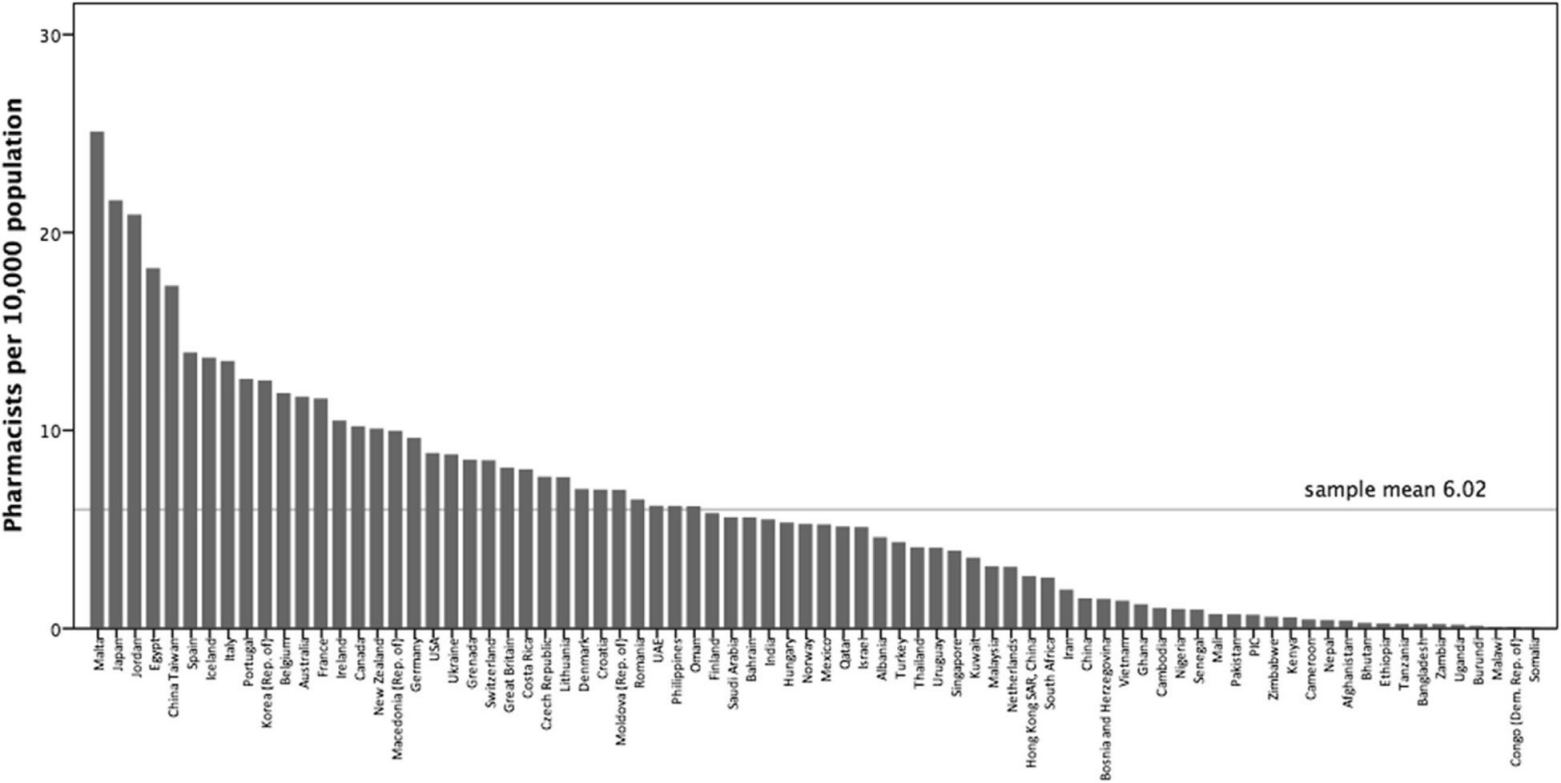
The density of pharmacists (per 10 000 population) displayed by country in descending order

To standardise the measure of pharmacists, an analysis of the population density of pharmacists (per 10 000 population) was performed (Fig. 1). The sample mean was 6.02 pharmacists per 10 000 population (*n* = 80). There is considerable variation between the surveyed countries/territories ranging from 0.02 (Somalia) to 25.07 (Malta) pharmacists per 10 000 population. African nations have significantly fewer pharmacists per capita.

**Fig. 2:**
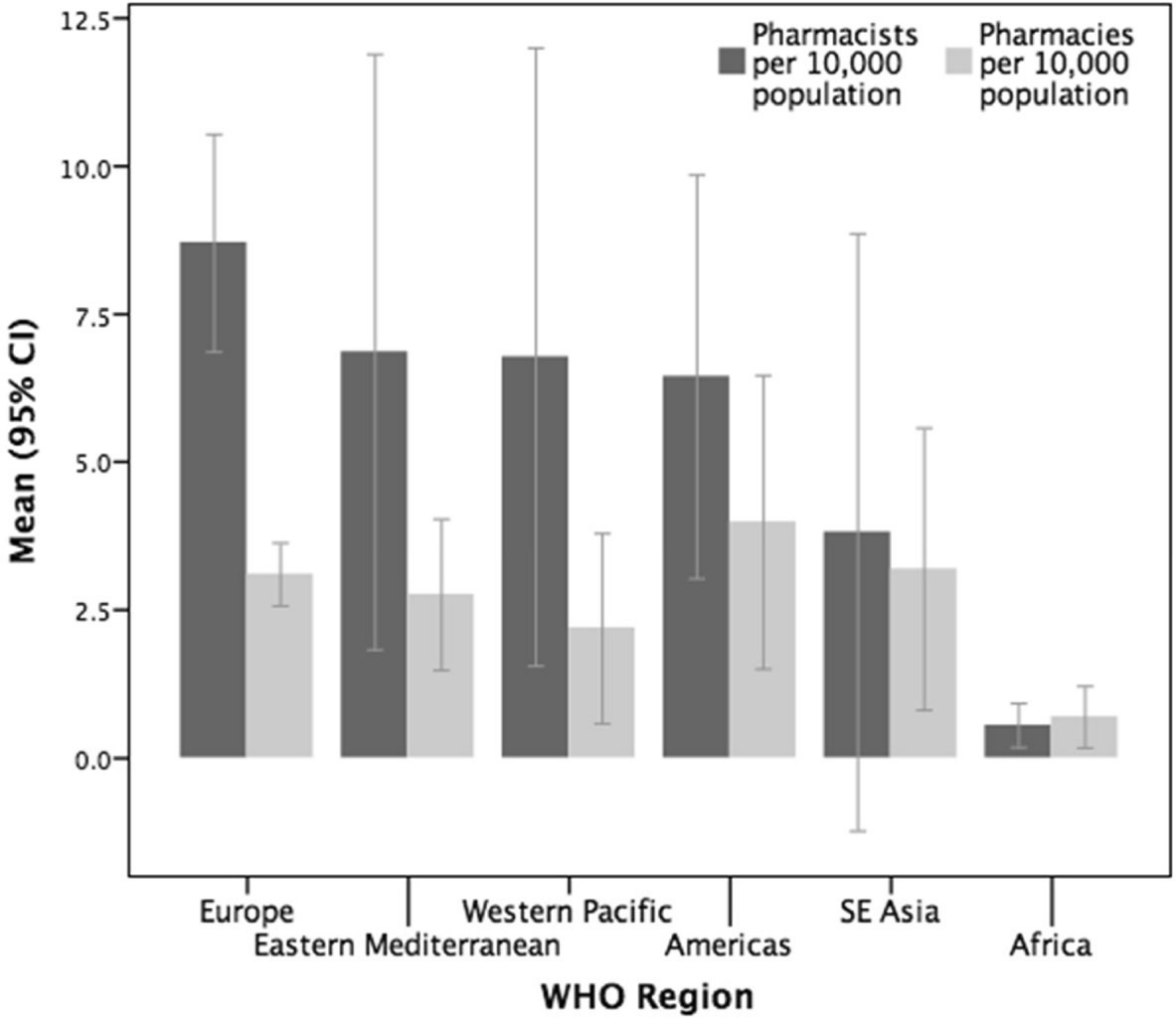
The mean densities of pharmacists and pharmacies displayed by WHO region (mean density per 10 000 population)

Through the comparative analysis of the pharmacist and pharmacy (community-based premises) densities, it is recognised that for all countries/territories involved in this survey, there is a greater number of pharmacists in contrast to pharmacies (Fig. 2). However, when comparing by WHO region, differences emerge with the Africa region which has more pharmacies than pharmacists per capita. Additionally, some countries report more pharmacies than pharmacists (Afghanistan, Bangladesh, Bhutan, Burundi, India, Nepal, Pakistan, Somalia, Vietnam).

There is a relationship between the economic status of a country (measured by gross national income (GNI) per capita), health expenditure per capita, and pharmacist density. Pharmacist density correlates with GNI and health expenditure (*r* = 0.48 *P* < 0.0001; *r* = 0.43 *P* < 0.0001, respectively).

**Fig. 3:**
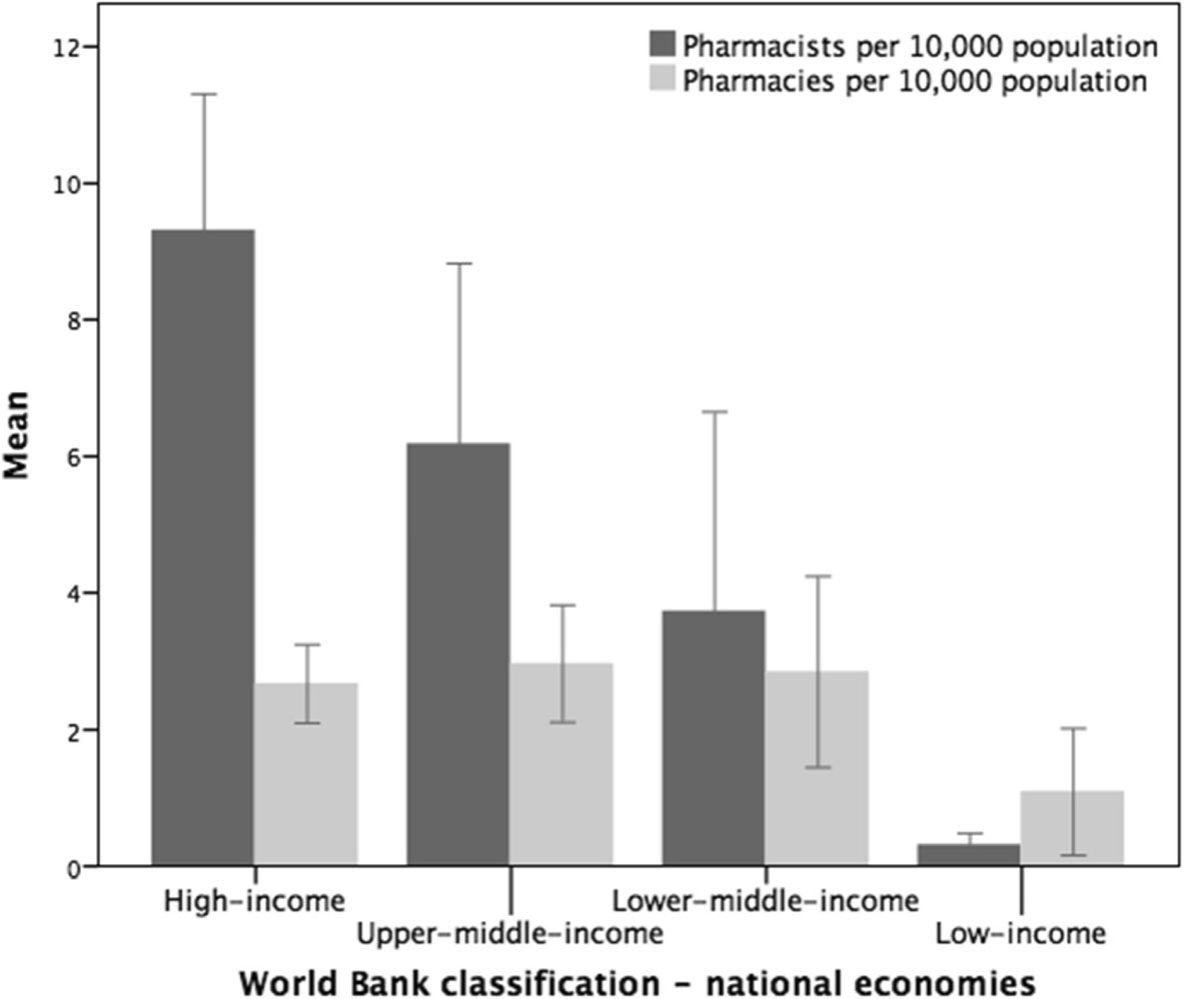
The mean densities of pharmacists and pharmacies displayed by World Bank economic classification (mean density per 10 000 population)

Mapping pharmacists per capita density with World Bank classification shows the relationship of the workforce with economic indicators (Fig. 3). There are demographic similarities with the lower-middle and upper-middle categories but large differences between low-income and high-income countries.

**Fig. 4:**
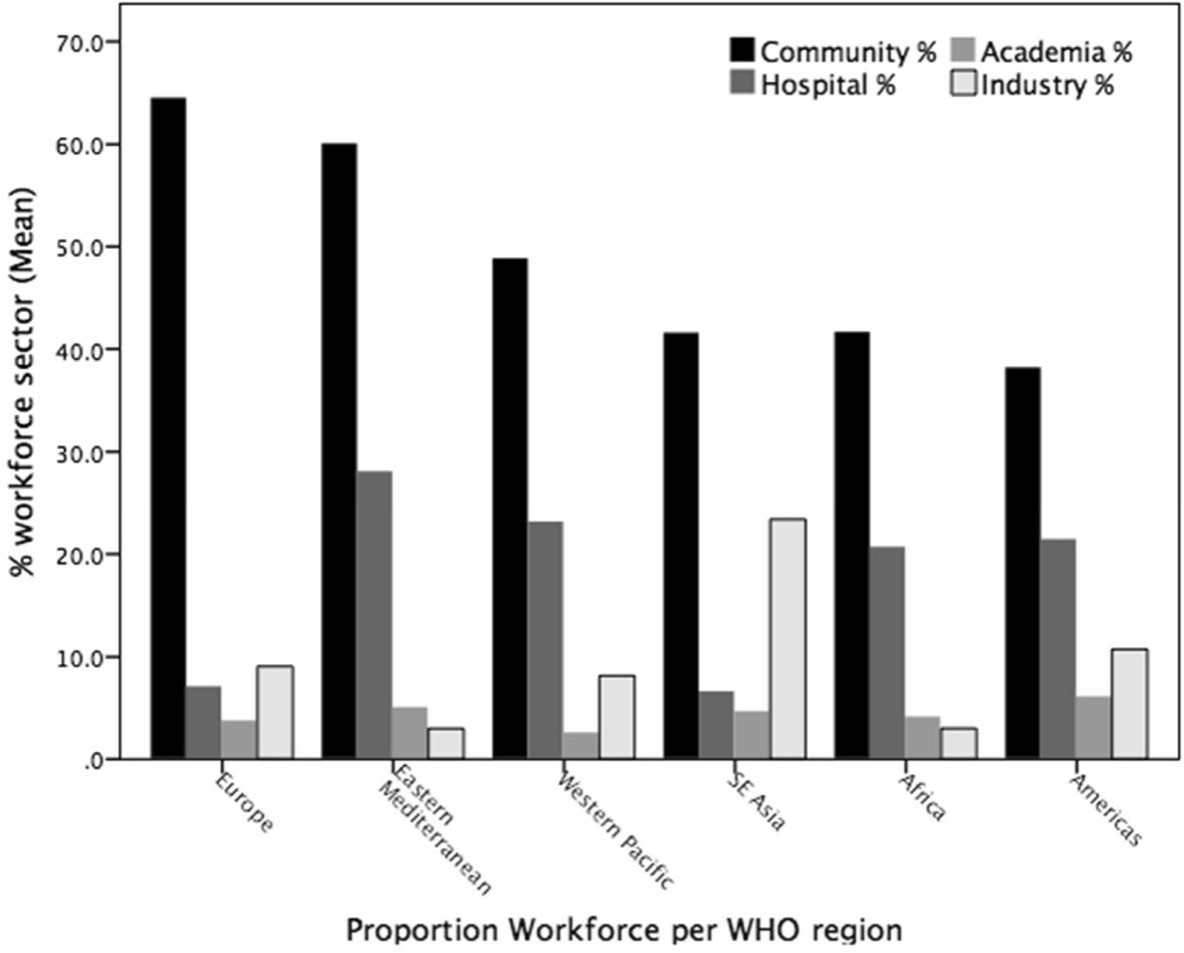
Proportions of pharmacist workforce (sector %) displayed by WHO region

The majority of pharmacists are employed in community pharmacy, followed by hospital, industry, research and academia, and regulation (Fig. 4). The distribution across sectors varies among countries though regional trends can be seen. The survey revealed on average 55 % of pharmacists worked in community pharmacies, 18 % in hospitals, 10 % in industry, 5 % in research and academia, and 5 % in regulation. Less than 5 % of the total pharmacist workforce in Africa is employed in the pharmaceutical industry. Conversely, the Southeast Asian region shows the proportion of industrial-sector employment of pharmacists is 30 %. The European region has the highest proportion of the pharmacy workforce working in the community settings.

**Fig. 5:**
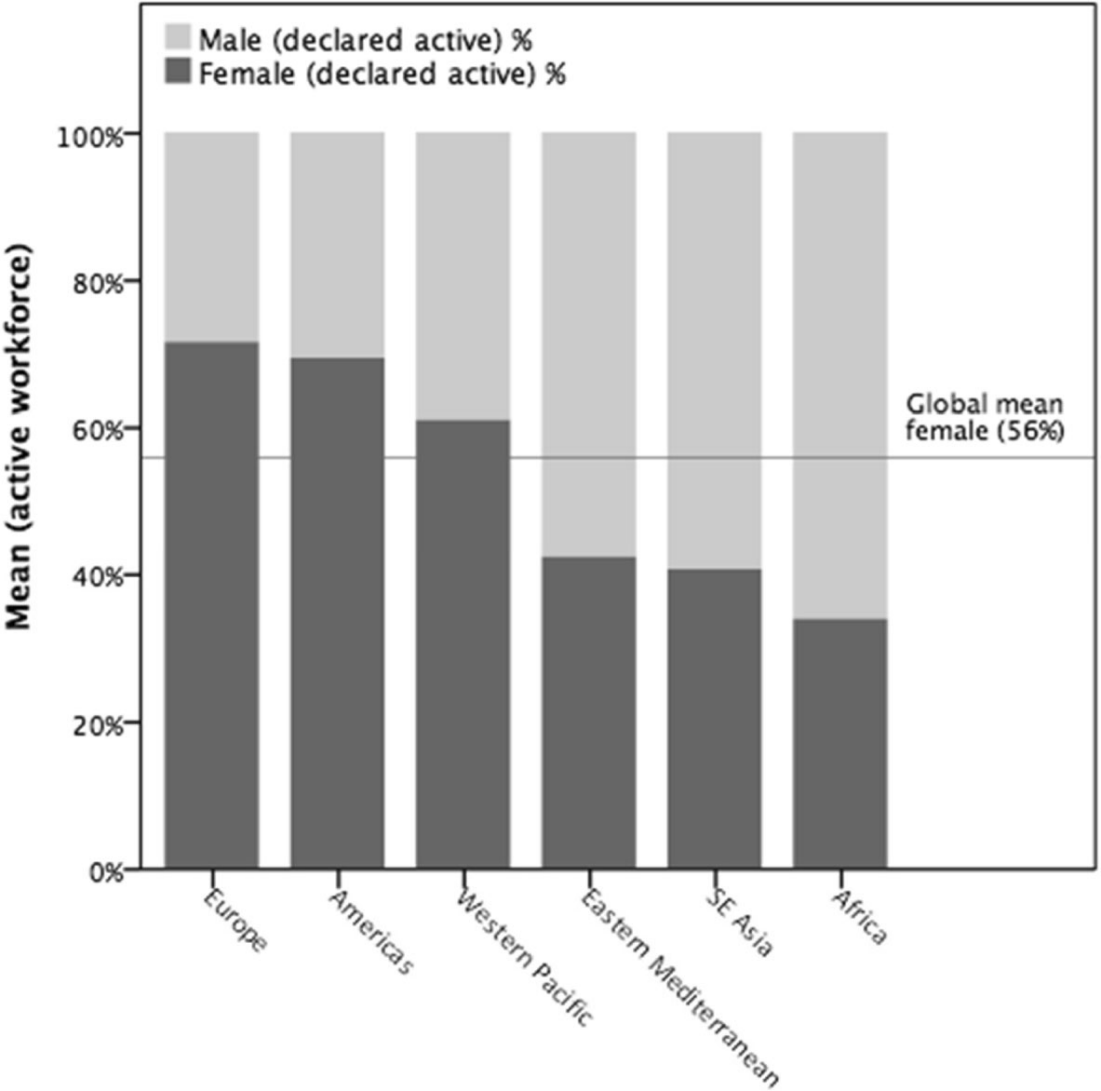
Proportion of pharmacist workforce displayed by gender and WHO region (%)

There is a greater proportion of females in the pharmacy workforce globally, with some WHO regions displaying female representation of more than 65 %. Overall, the survey shows consistent trends that the number of female pharmacists entering the profession is increasing. However, at a country and WHO region level, this distribution is varied (Fig. 5).

## Discussion

There is a wide variability in the pharmacist workforce and in the supply of pharmacists globally between countries. Pharmacy workforce per capita varies considerably between countries and regions and generally correlates with the size of population. Globally, countries with lower numbers of pharmacists per capita are likely to have less access to medicines as well as provision of pharmaceutical services and advice.

This study provides an assessment of the workforce stock of pharmacists in the countries who responded to the survey. It is not possible to draw full conclusions on whether there is a sufficient supply of pharmacists with the relevant competencies and skill mix. For those countries with low densities of pharmacists, it is possible that there is a gap between the need for pharmacy services and the actual supply of pharmacists. Demand indicators based on disease burden/need for services would help assess if gaps are present. Pharmacist density is not an end in itself but a means to improve health. Absolute numbers of pharmacists do not reflect the issue of part-time workers and their proportion of the workforce. Nor does density of pharmacists describe the productivity of the workforce. There may also be differences in the urban/rural distribution of pharmacists and other factors may impact on a nation’s supply of pharmacists (e.g. healthcare policies and systems) that are beyond the scope of this paper.

Figure 2 raises the issue of whether there is appropriate supervision of pharmaceutical services in those countries that have more pharmacies (premises) than pharmacists. Additionally, African countries in general lack both pharmacies and pharmacists, which again has implications for access to medicines due to lack of availability (and likely inequitable distribution) of access points—as well as a skilled workforce to give pharmaceutical advice. Those countries with more pharmacies than pharmacists may be using other staff, for example, pharmacy technicians, pharmacy support workers, nurses, or others as the workforce, and further research is required to ascertain if this is the case.

The correlation between economic status of a country (as measured by GNI per capita) and related health expenditure per capita with pharmacist density implies that expenditure on health and pharmacist availability directly relate to economic development.

The results showed that there is a linear association with standardised pharmacist numbers and World Bank classification (Fig. 3). The gap increases between pharmacists and pharmacies with economic income, perhaps due to greater employment opportunities for pharmacists in high-income countries. In addition, the density of pharmacies is greater than that of pharmacists in low-income countries and territories, which suggests difficulty in access to medicines in these environments.

It is likely that the distribution of pharmacists by sector within all regions, with community pharmacy being the largest, reflects the model of delivery of pharmaceutical care and access to medicines in individual nations; for most countries, community-based pharmaceutical health services are the main form of provision with hospitals providing more pharmaceutical specialist care (Fig. 4). (A community pharmacy is defined as a pharmacy providing access to medicines for a specific community (often retail premises). A hospital pharmacy is one which is located in a hospital.)

The increase in the proportion of females in the workforce suggests that pharmacy remains an attractive career for women. This may be due to evolving roles such as increased patient-facing duties as well as greater flexibility of career structures and breaks.

## Conclusions

Analysing and monitoring the status of the pharmacy workforce is important if workforce challenges are to be addressed and workforce risks mitigated. However, there is no universal coverage of pharmacy workforce intelligence—human resource information systems are still weak in many countries, and the number of countries publishing regular and consistent pharmacy workforce data is considered to be low.

Pharmacy workforce density varies considerably between countries and workforce regions and generally correlates with population numbers and country-level economic indicators. Those countries and territories with lower economic indicators tend to have fewer pharmacists and pharmacy technicians—this has implications for inequalities in access to medicines and medicine expertise. Ongoing efforts are needed to ensure capacity building of skilled medicine expertise to meet the pharmaceutical needs of populations.

## Abbreviations

FIP: International Pharmaceutical Federation
FIP*Ed*: FIP Education Initiative
GNI: Gross national income
MDGs: Millennium Development Goals
PICs: Pacific Island Countries
WHO: World Health Organization
